# The characteristics of arterial spin labeling cerebral blood flow in patients with subjective cognitive decline: The Chinese imaging, biomarkers, and lifestyle study

**DOI:** 10.3389/fnins.2022.961164

**Published:** 2022-08-02

**Authors:** Wenyi Li, Jiwei Jiang, Xinying Zou, Yuan Zhang, Mengfan Sun, Ziyan Jia, Wei Li, Jun Xu

**Affiliations:** ^1^Department of Neurology, Beijing Tiantan Hospital, Capital Medical University, Beijing, China; ^2^China National Clinical Research Center for Neurological Diseases, Beijing, China

**Keywords:** subjective cognitive decline, dementia, cerebral blood flow, neuroimaging, parahippocampal gyrus

## Abstract

**Objective:**

We aimed to characterize the potential risk factors and cerebral perfusion of patients with subjective cognitive decline (SCD).

**Methods:**

This prospective study enrolled consecutive patients from the Chinese Imaging, Biomarkers, and Lifestyle (CIBL) Cohort of Alzheimer’s disease between February 2021 and March 2022. Patients who met the SCD diagnostic criteria were categorized into the SCD group, while those without cognitive complaints or any concerns were assigned to the healthy control (HC) group. The demographic and clinical characteristics and cerebral blood flow (CBF) from pseudo-continuous arterial spin labeling (pCASL) in standard cognitive regions were compared between these two groups. A multivariate analysis was performed to identify independent factors associated with SCD.

**Results:**

The frequency of family history of dementia in the SCD group was higher compared with the HC group (*p* = 0.016). The CBF of left hippocampus (*p* = 0.023), left parahippocampal gyrus (*p* = 0.004), left precuneus (*p* = 0.029), left middle temporal gyrus (*p* = 0.022), right parahippocampal gyrus (*p* = 0.018), and right precuneus (*p* = 0.024) in the SCD group were significantly increased than those in the HC group. The multivariate logistic regression analyses demonstrated that the family history of dementia [*OR* = 4.284 (1.096–16.747), *p* = 0.036] and the CBF of left parahippocampal gyrus [*OR* = 1.361 (1.006–1.840), *p* = 0.045] were independently associated with SCD.

**Conclusion:**

This study demonstrated that the family history of dementia and the higher CBF within the left parahippocampal gyrus were independent risk factors associated with patients with SCD, which could help in the early identification of the SCD and in intervening during this optimal period.

## Introduction

With the global increase in the elderly population, Alzheimer’s disease (AD), the leading cause of dementia in the elderly, has been the greatest challenge for global public health and social care. The Alzheimer’s Disease International (ADI) predicted that the global population suffering from dementia would rise to 82 million in 2030 and exceed 152 million in 2050 (Patterson, 2018). However, having limited options for assessing the exact pathophysiology and early diagnosis of AD, the clinical experts are faced with great challenge in providing effective therapeutic strategies (Selkoe, 2021). Current drugs for AD mainly aim at the correction of neurotransmitter abnormalities, such as cholinesterase enzyme inhibitors (ChEIs) and *N*-methyl D-aspartate (NMDA) receptor antagonists, which can only provide symptomatic treatment to the disease to a certain extent but cannot reverse the disease progression (Joe and Ringman, 2019).

In 2018, the National Institute on Aging and Alzheimer’s Association (NIA-AA) proposed the “ATN” research framework, including biomarkers of Aβ deposition, pathologic tau, and neurodegeneration, which emphasized the progression of AD as a continuum (Jack et al., 2018). Asymptomatic individuals with abnormal AD biomarkers should be considered as a stage of AD, called “preclinical AD,” rather than a separate diagnosis. The Dominantly Inherited Alzheimer Network (DIAN) study has demonstrated that in the continuous progress, longitudinal Aβ begins to change first (starting 25 years before estimated symptom onset), followed by the declines in cortical metabolism and CSF p-tau_181_ and tau (approximately 7–10 years later), then the cognitive decline and the hippocampal atrophy (approximately 20 years later) (McDade et al., 2018). In preclinical AD, individuals still retain sufficiently intact cognitive function that can be harnessed and directed toward either compensation or restitution of function (Rabin et al., 2017). Thus, the preclinical stage should be an important consideration for starting the interventions to prevent cognitive impairment before the onset of clinical symptoms.

Subjective cognitive decline (SCD) refers to the self-perception of cognitive decline and does not require confirmation by external observation (Jessen et al., 2014). SCD is a generalized heterogeneous concept that can be induced by many conditions other than AD (Jessen et al., 2020). Increasing evidence has demonstrated that SCD in the elderly is a risk factor for mild cognitive impairment (MCI) or dementia (van Harten et al., 2018; Jessen et al., 2020; Pike et al., 2021). Moreover, abnormalities in several AD-related biomarkers in cerebrospinal fluid or neuroimaging were also found in individuals with SCD (Slot et al., 2019; Jessen et al., 2020), which makes SCD the second stage of six numeric stages in the AD continuum. Thus, effective recognition of SCD may provide important clues for a preclinical stage closely related to dementia or AD. It has been widely accepted that modifying risk factors might prevent or delay up to 40% of dementias (Livingston et al., 2020), which highlights the feasibility and importance of early prevention. However, the implementation of the “ATN” framework has limitations due to the invasive of lumbar puncture and the expense of positron emission tomography (PET). The SCD that is being recognized clinically still needs to be paired with other feasible biomarkers for improving its diagnostic value.

In addition to Aβ deposition, recent evidence from neuroimaging cohort studies and animal models strongly suggests other underlying pathophysiological processes of AD, particularly neurovascular dysregulation (Iturria-Medina et al., 2016; Dounavi et al., 2021). Vascular dysfunction result in reduced clearance of Aβ by periarteriolar and impaired Aβ transporters across the blood-brain barrier, which can increase Aβ deposition. Aβ induces contraction of pericytes and vascular smooth muscle cells and then exacerbates hypoperfusion (Fisher et al., 2022). Duan et al. (2020) have proposed that CBF could be a neuroimaging marker to reflect the degree of cognitive impairment. Arterial spin labeling (ASL) is a non-invasive technique for quantifying cerebral perfusion that has been proven as a useful biomarker of the early stages of AD, which is consistent with the findings of PET (Hays et al., 2016). However, despite increasing studies of ASL on dementia, only a few on individuals with SCD are available, and the findings seem to be not in agreement. Hays et al. (2018) have demonstrated significant negative correlations between verbal memory and CBF within the posterior cingulate cortex, middle temporal gyrus, hippocampus, fusiform gyrus, and inferior frontal gyrus in patients with SCD. Another observational study has revealed that compared with elderly controls, participants with SCD presented a significant decline in CBF values, mainly in the hippocampal and posterior cingulate cortex (Yang et al., 2021). Moreover, the outstanding relevance of classical risk factors for dementia may not be proven in SCD, which makes SCD difficult to be identified (Wen et al., 2021). The significant clinical and neuroimaging characteristics of SCD are yet to be identified. Therefore, our study aimed to explore risk factors of SCD, especially the perfusion characteristics, for providing new clues to early recognition and intervention of SCD.

## Materials and methods

### Ethics

The study was approved by the Institutional Review Board of Beijing Tiantan Hospital of Capital Medical University (KY-2021-028-01) before enrolling participants. All individuals involved in this study provided written informed consent for clinical and genetic analyses before enrollment.

### Patient or participant selection

All data were analyzed from the Chinese Imaging, Biomarkers, and Lifestyle (CIBL) study of AD, an ongoing large-scale prospective cohort study majorly conducted in 2020 and focused on the risk factors, biomarkers, and neuroimaging in the Chinese population with cognitive impairment, which is registered at chictr.org.cn (ChiCTR2100049131). This study selected consecutive patients with SCD between February 2021 and March 2022. The inclusion criteria were as follows: (1) SCD that met the diagnostic framework proposed by the SCD Initiative Working Group in 2014 and 2020 (Jessen et al., 2014; Jessen et al., 2020); (2) self-experienced persistent decline in memory, rather than other domains of cognition, while healthy control (HC) volunteers with no cognitive complaints or any concerns (worries); (3) normal performance on standardized cognitive tests, adjusted for age, gender, and years of education; and (4) onset of SCD within the last 5 years. Patients were excluded from the study if they (1) were left-handed/ambidexter; (2) met the criteria for MCI or dementia; (3) had other central nervous system diseases that may cause cognitive impairment, such as stroke, Parkinson’s disease, frontotemporal dementia, tumor, encephalitis, and epilepsy; (4) had a mental disorder history that met the Diagnostic and Statistical Manual of Mental Disorders-5 (DSM-5); (5) presented cognitive impairment due to traumatic brain injury; (6) had a history of drug abuse or toxic exposure; (7) had systemic diseases, such as thyroid dysfunction, syphilis, and HIV; and (8) had congenital mental developmental delay. Patients with SCD were categorized into the SCD group, while those who met HC volunteers were assigned to the control group.

### Data collection

Information on sex, age of enrolled in the cohort, years of education, medical history, family history of dementia, and clinical symptoms were collected. DNA samples were extracted from whole blood samples. *Apolipoprotein E (APOE)* genotyping was performed based on two single nucleotide polymorphism (SNP) sites (rs429358 and rs7412) at WeGene Lab using a customized Illumina WeGene V3 Array by Illumina iScan System, which contains roughly 700,000 markers. All participants completed a comprehensive neuropsychological assessment to evaluate their global cognitive functions, such as Mini-mental State Examination (MMSE), the Montreal Cognitive Assessment (MoCA), the Hamilton Depression Rating Scale (HAMD), and the Hamilton Anxiety Rating Scale (HAMA). The SCD-questionnaire 9 (SCD-Q9) was performed to help diagnose and quantitatively assess the severity of SCD (Gifford et al., 2015; Hao et al., 2017). In this study, two trained neurologists or neuropsychologists performed the cognitive tests, respectively.

### Magnetic resonance imaging acquisition and processing

Image acquisition was performed on a 3-T MR scanner (SIGNA Premier; GE Healthcare, Milwaukee, WI, United State) with a 48-channel head coil. High-resolution 3D T1 scans were performed using the Inversion Recovery Gradient Recalled Echo (IR-GRE) sequence with the following parameters: repetition time (TR) = 7.3 ms, echo time (TE) = 3.0 ms, inversion time (TI) = 450 ms; flip angle (FA) = 12 degrees, field of view (FOV) = 256 mm × 256 mm, acquisition matrix = 256 × 256, slice thickness = 1.0 mm, slice number = 176, and scan time = 4 min 56 s. ASL was performed using pseudo-continuous arterial spin labeling (pCASL) with a 3D readout (3D pCASL) sequence with the following parameters: axial acquisition, TR = 4,849 ms, TE = 10.6 ms, FOV = 220 mm × 220 mm, acquisition matrix = 512 × 512, slice thickness = 4 mm, slice number = 36, post-labeling delay = 2,025 ms, and scan time = 4 min 22 s.

Data processing was performed using CereFlow software (Anying Technology Beijing Co., Ltd.)^1^ with the following steps: (1) calculation of CBF from the GE scanner’s ASL’s perfusion-weighted (PW) image and proton density (PD) image using the standard simple compartment model with an assumption that the arterial transit time (ATT) is equivalent to post-label delay (PLD); (2) co-registration of the M0 image (GE ASL’s PD image) with the anatomical T1w image, the calculated CBF image was also co-registered to T1w with the same transformation parameters; (3) normalization of T1w images to the Montreal Neurological Institute (MNI) template; (4) CBF image warped into the MNI space using the forward transformation matrix derived from T1w; and (5) extraction of the regional CBF by the automated anatomical labeling (AAL) atlas (Tzourio-Mazoyer et al., 2002). The brain areas that showed significant CBF differences between the two groups were further analyzed, including bilateral hippocampal, parahippocampal gyrus, precuneus, middle temporal gyrus, and posterior cingulate.

### Statistical analysis

All statistical analyses were performed using SPSS 26.0 statistical software (SPSS Inc., Chicago, IL, United States). Categorical variables are presented as the total number (*n*) and percentage (%) per group, and the χ^2^ or Fisher’s exact test was used to assess statistical differences. The mean and standard deviations (SDs) were calculated for continuous variables with normal distribution, while the median and interquartile range (IQR) were used for continuous variables lacking a normal distribution. Subsequently, the *t*-test was used for normally distributed data. The Mann–Whitney *U*-test was used for data with no normal distribution. Risk factors (*p* < 0.05) were further analyzed using univariate and multivariate logistic regression. The *p*-values smaller than 0.05 were considered statistically significant.

## Results

### Baseline characteristics in both groups

**FIGURE 1:**
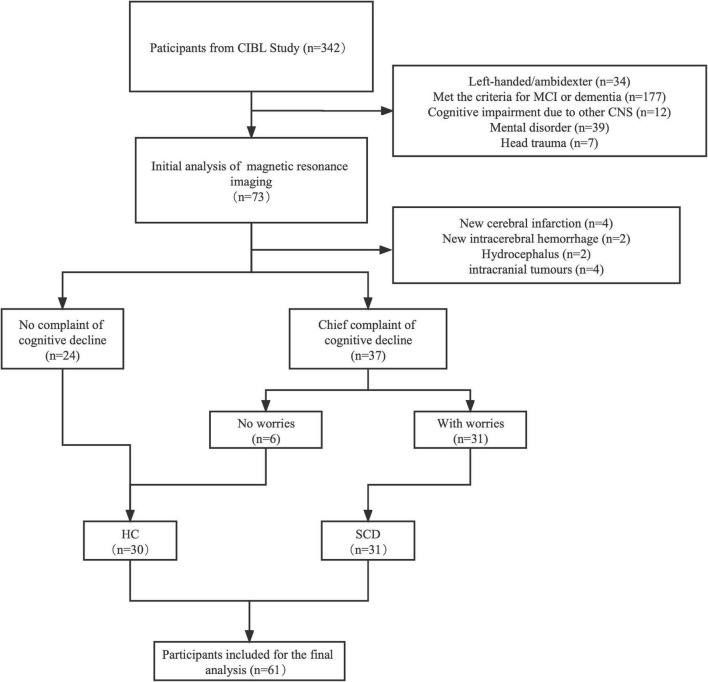
A flowchart of the inclusion and exclusion criteria. MRI, magnetic resonance imaging; MCI, mild cognition impairment; HC, healthy control; SCD, subjective cognition decline.

The clinical variables and demographics are shown in Table 1. In the initial stage, a total of 349 participants underwent basic information collection and neuropsychological assessment, and then 61 patients were included in the final analysis (Figure 1). Of these, 31 patients (50.8%) were diagnosed with SCD and 30 patients (49.2%) were assigned to the control group. There was no significant difference between the two groups in gender, age, education, and the prevalence of hypertension and diabetes, except for a family history of dementia. The frequency of family history of dementia in the SCD group (45.2%) was significantly higher than that in the HC group (16.7%). Subsequently, we compared the scores of MMSE, MoCA, HAMD, and HAMA and found that there was no significant difference between the two groups in the neuropsychological assessments. Additionally, we found no significant difference in the frequency of *APOE* ε4 carriers between the two groups.

**TABLE 1:** Demographic and neuropsychological assessments for participants.

Variables	HC (*n* = 30)	SCD (*n* = 31)	*p*-value
Age (years)	62.53 ± 7.71	60.94 ± 9.33	0.496
Gender (%, Male)	12 (40)	7 (22.6)	0.142
Education (years)	10.60 ± 4.70	12.68 ± 3.52	0.056
Hypertension (%)	13 (43.3)	7 (22.6)	0.084
Diabetes (%)	7 (23.3)	2 (6.5%)	0.081
Family history of dementia (%)	5 (16.7)	14 (45.2)	**0.016**
MMSE (scores)	29 (27.75–29)	29 (28–29)	0.703
MoCA (scores)	26 (23.75–28)	27 (26–28)	0.090
HAMD (scores)	4 (1–7)	6 (3–8)	0.171
HAMA (scores)	3 (0.75–5.5)	4 (1–6)	0.301
*APOE* genotype (%)			0.603
2/3	8 (26.7)	5 (16.1)	
3/3	17 (56.7)	20 (64.5)	
3/4	5 (16.7)	6 (19.4)	

MMSE, Mini-mental State Examination; MoCA, the Montreal Cognitive Assessment; HAMD, Hamilton Depression Rating Scale; HAMA, Hamilton Anxiety Rating Scale; HC, healthy control; SCD, subjective cognitive decline. The numbers in bold are statistically significant (p-value 0.05).

### The absolute cerebral blood flow in both groups

**FIGURE 2:**
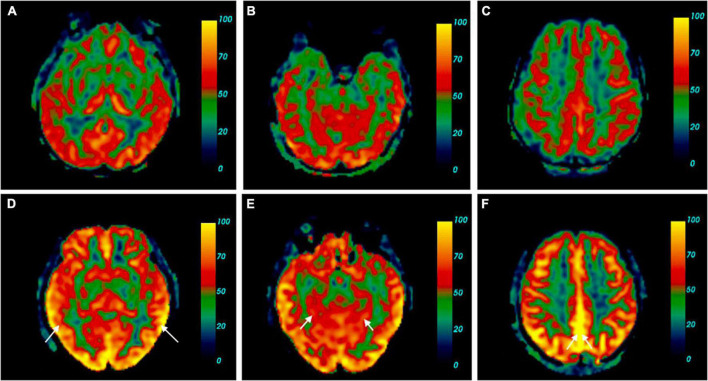
Cerebral perfusion map contrast of SCD and HC. **(A–C)** Were cerebral blood flow maps from one of the HC. **(D–F)** Belong to a participant with SCD [female, 60 years old, complaint of memory decline for 2 years, sister has a history of dementia, mini-mental state examination (MMSE) 30, the Montreal Cognitive Assessment (MoCA) 29, SCD-Q9 6, and *Apolipoprotein E (ApoE)* ε3/ε4]. Brain pseudo-continuous arterial spin labeling (pCASL) revealed the patient with SCD had increased cerebral perfusion, especially in the bilateral middle temporal gyrus **(A)**, the absolute CBF in the right bilateral middle temporal gyrus of the HC was 42.20 ml/100 g/min, the left was 37.49 ml/100 g/min. **(D)** The absolute CBF in the right bilateral middle temporal gyrus of the SCD was 67.69 ml/100 g/min, the left was 74.47 ml/100 g/min, parahippocampal gyrus **(B)**, the absolute CBF in the right parahippocampal gyrus of the HC was 27.59 ml/100 g/min, the left was 35.27 ml/100 g/min. **(E)** The absolute CBF in the right parahippocampal gyrus of the SCD was 50.99 ml/100 g/min, the left was 53.73 ml/100 g/min, precuneus **(C)**, the absolute CBF in the right precuneus of the HC was 39.44 ml/100 g/min, the left was 43.15 ml/100 g/min. **(F)** The absolute CBF in the right precuneus of the SCD was 71.42 ml/100 g/min, the left was 77.33 ml/100 g/min.

The absolute CBF in various brain regions between the two groups is shown in Table 2. The CBF of the SCD group was generally higher than the HC group, and the differences in six brain regions, including the left hippocampus (*p* = 0.023), left parahippocampal gyrus (*p* = 0.004), right parahippocampal gyrus (*p* = 0.018), left precuneus (*p* = 0.029), right precuneus (*p* = 0.024), and left middle temporal gyrus (*p* = 0.022) were statistically significant. The comparison of the cerebral blood perfusion diagram in Figure 2 also conforms to the above statistics.

**TABLE 2:** Differences in CBF of cognition-related brain regions between the two groups.

Brain regions	CBF (ml/100 g/min)		*p*-value
	HC (*n* = 30)	SCD (*n* = 31)	
Hippocampus_L	40.96 ± 8.20	45.98 ± 8.64	**0.023**
Hippocampus_R	40.79 ± 8.60	44.80 ± 8.03	0.065
Posterior cingulate_L	62.05 ± 11.08	69.05 ± 17.24	0.064
Posterior cingulate_R	52.99 ± 10.85	58.62 ± 14.83	0.097
Parahippocampal gyrus_L	37.60 ± 6.48	42.52 ± 6.15	**0.004**
Parahippocampal gyrus_R	39.54 ± 7.67	44.19 ± 7.32	**0.018**
Precuneus_L	50.62 ± 10.41	56.85 ± 11.33	**0.029**
Precuneus_R	50.66 ± 10.55	57.06 ± 11.08	**0.024**
Middle temporal gyrus_L	53.04 ± 9.46	59.30 ± 11.27	**0.022**
Middle temporal gyrus_R	48.74 ± 8.55	52.69 ± 9.28	0.089

CBF, cerebral blood flow; HC, healthy control; SCD, subjective cognitive decline. The numbers in bold are statistically significant (p-value 0.05).

### Univariate and multivariate logistic regression analyses

We assessed all risk factors with *p* values < 0.05 (Tables 1, 2) using a univariate and multivariate logistic regression model. The results of univariate logistic regression analyses were consistent with the *T*-test and the *χ^2^* test (Table 3), which showed that the family history of dementia and CBF in the above-mentioned brain regions were significantly increased in the SCD group compared with the HC group. According to the odds ratio (*OR*) value, the first five factors were selected for multivariate logistic regression analysis. The multivariate logistic regression analyses demonstrated that the family history of dementia [*OR* = 4.284 (1.096–16.747), *p* = 0.036] and the CBF of left parahippocampal gyrus [*OR* = 1.361 (1.006–1.840), *p* = 0.045] were independently associated with SCD (Table 4).

**TABLE 3:** Univariate logistic regression analyses of risk factors in patients with SCD.

Variables	B	SE	Wald	OR	95% CI	*p*-value
Family history of dementia	1.415	0.608	5.410	4.118	1.249–3.570	**0.020**
Hippocampus_L	0.074	0.034	4.736	1.076	1.007–1.150	**0.030**
Hippocampus_R	0.060	0.033	3.277	1.061	0.995–1.132	0.070
Posterior cingulate_L	0.035	0.019	3.226	1.035	0.997–1.075	0.072
Posterior cingulate_R	0.035	0.021	2.671	1.035	0.993–1.079	0.102
Parahippocampal gyrus_L	0.124	0.046	7.351	1.132	1.035–1.238	**0.007**
Parahippocampal gyrus_R	0.085	0.038	5.072	1.089	1.011–1.173	**0.024**
precuneus_L	0.054	0.025	4.441	1.055	1.004–1.109	**0.035**
precuneus_R	0.056	0.026	4.655	1.058	1.005–1.114	**0.031**
Middle temporal gyrus_L	0.060	0.027	4.771	1.061	1.006–1.120	**0.029**
Middle temporal gyrus_R	0.051	0.030	2.813	1.052	0.991–1.117	0.093

SE, standard error; OR, odds ratio; CI, confidence interval. The numbers in bold are statistically significant (p-value 0.05).

**TABLE 4:** Multivariate logistic regression analyses of risk factors in patients with SCD.

Factors	B	SE	Wald	OR	95% CI	*p*-value
Family history of dementia	1.455	0.696	4.375	4.284	1.096–16.747	**0.036**
Hippocampus_L	–0.105	0.084	1.551	0.901	1.006–1.840	0.213
Parahippocampal gyrus_L	0.308	0.154	4.002	1.361	1.006–1.840	**0.045**
Parahippocampal gyrus_R	0.006	0.103	0.003	1.006	0.822–1.230	0.956
precuneus_R	−0.018	0.068	0.074	0.982	0.860–1.121	0.786
Middle temporal gyrus_L	−0.040	0.069	0.345	0.961	0.840–1.099	0.557

SE, standard error; OR, odds ratio; CI, confidence interval. The numbers in bold are statistically significant (p-value 0.05).

## Discussion

Currently, there is no consensus on which clinical and neuroimaging characteristics of individuals lead to a higher risk of developing SCD, despite the increasing research evidence about the clinical and cerebral microstructural and blood flow alterations associated with SCD (Yang et al., 2021; Zhang et al., 2021). We speculate that the different findings may be related to the ethnic difference, different inclusion criteria, and factors analysis methods. This study demonstrated that the family history of dementia and the hyperperfusion alterations in the left parahippocampus were independent risk factors associated with patients with SCD, which could help in the early identification of the SCD and in intervening during this optimal period. The results of Wolfsgruber et al. (2022) are consistent with ours, which found that the first-degree family history of AD revealed higher SCD-plus scores than healthy controls. Another prospective cohort study has found that greater subjective memory impairment is associated with a first-degree family history of AD in healthy older adults (Haussmann et al., 2018). As we all know, the uncontrollable and common risk factors of dementia include aging, a first-degree family history of dementia, carrying *APOE* ε4 allele, and being a female, especially after the age of 80 years; among these, the strongest risk factors are advanced age and *APOE* ε4 allele carrier (Scheltens et al., 2021). However, there were no significant differences of *APOE* ε4 allele and aging between patients with SCD and those elderly controls in this study. A previous study found that neither the family history of dementia nor *APOE* ε4 status was associated with SCD (Nicholas et al., 2017). The above-mentioned evidence suggested that SCD was highly heterogeneous, and the classical risk factors for AD and SCD might be mismatched (Wen et al., 2021). Therefore, it is not enough to use AD-related risk factors alone to evaluate and predict SCD.

One of the key points in our study is the measurement of CBF via ASL. ASL is an MRI perfusion technique that enables quantification of the CBF of cerebral regions without the need for contrast injection. Compared with the Aβ-PET or tau-PET, ASL is more widely used in clinical practice and research, and with ^18F^FDG PET/CT, ASL is less expensive without ionizing radiation exposure (Dolui et al., 2020). Recently, ASL has been performed for patients with cognitive impairment, which is believed to closely match between components, regional and quantitative hypoperfusion and hypometabolism by ^18F^FDG PET (Ho, 2018; Riederer et al., 2018). In 2015, the ISMRM Perfusion Study Group and the European ASL in Dementia Consortium released consensus guidelines that recommended the standardized implementation of 3D pseudo-continuous ASL (pCASL) with background suppression (Alsop et al., 2015). In a recent head-to-head comparison, the multiplanar and multi-delay pCASL on a GE Signa PET/MR have similar diagnostic accuracy in dementia to the ^18F^FDG PET (Ceccarini et al., 2020). The evidence suggests significant diagnostic and application value of pCASL in the cognitive impairment disorders.

However, to the best of our knowledge, only a few pCASL studies of individuals with SCD are available, and the findings are inconsistent. Hays et al. (2018) demonstrated that patients with SCD displayed higher CBF in the posterior cingulate cortex, middle temporal gyrus, hippocampus, fusiform gyrus, and inferior frontal gyrus. Another study that investigated regional CBF in 162 Alzheimer’s Disease Neuroimaging Initiative participants had shown that patients with SCD had increased hippocampal and inferior parietal CBF relative to HC participants (Thomas et al., 2021). The hyperperfusion within the classical cognitive areas may reflect early neurovascular dysregulation, whereby higher CBF is needed to maintain tissue metabolism and cognitive functioning and is also reflective of early cognitive inefficiencies that distinguish SCD from healthy elderly people (Østergaard et al., 2013). Our study revealed that increased CBF in the left parahippocampus was independent risk factor associated with SCD, which was rarely described before in SCD. Choo et al. (2019) have found that subjective memory complaints were associated with Aβ depositions, especially in the left parahippocampus (Choo et al., 2019). There is evidence suggesting that parahippocampus volume reduction and thinning might reflect the initial sign of olfactory impairment and lead to dysfunction in the connection of olfactory memory to the neocortex (Kubota et al., 2020). These findings indicate that the left parahippocampal gyrus may be one of the earliest involved areas of cognitive decline. However, a prospective cohort study displayed that patients with SCD showed a poor level of CBF in the right middle frontal gyrus compared with the HC subjects (Zhang et al., 2021). Participants with SCD plus demonstrated a significant decline in CBF values, mainly in the hippocampal head and posterior cingulate cortex (Yang et al., 2021). Moreover, the study from the Amsterdam Dementia Cohort found that reduced CBF was not associated with worse performance in patients with SCD (Leeuwis et al., 2017). We speculate these controversial results may be due to the use of different cohorts or inclusion criteria (SCD or SCD plus) and image processing methodologies.

Our study has several limitations. This was a single-center study, and included a relatively limited number of individuals. Thus, the findings may not be applicable to other settings due to the inherent selection bias.

## Conclusion

This study found the family history of dementia and the hyperperfusion in the left parahippocampal gyrus were independently associated with SCD patients, which combined the risk factors of AD and evidence of vascular dysregulation for earlier identifying the SCD and interfering in this optimal period. Prospective multicenter studies are needed to evaluate the effectiveness of this finding and develop a reliable predictive model for SCD.

## Data availability statement

The original contributions presented in this study are included in the article/supplementary material, further inquiries can be directed to the corresponding author.

## Author contributions

WyL and JJ designed the study, analyzed the data, and drafted the manuscript. XZ, YZ, MS, ZJ, and WL edited and reviewed the manuscript. JX designed the study, revised the manuscript, and provided funding supports. All authors contributed to the article and approved the submitted version.

## Footnotes

1
www.cereflow.cn
